# Chemical hybridization of sulfasalazine and dihydroartemisinin promotes brain tumor cell death

**DOI:** 10.1038/s41598-021-99960-z

**Published:** 2021-10-21

**Authors:** Annemarie Ackermann, Aysun Çapcı, Michael Buchfelder, Svetlana B. Tsogoeva, Nicolai Savaskan

**Affiliations:** 1 Translational Cell Biology and Neurooncology Lab, Department of Neurosurgery, Universitätsklinikum Medical School Erlangen, Friedrich Alexander University of Erlangen – Nürnberg (FAU), Erlangen, Germany; 2 Organic Chemistry Chair I and Interdisciplinary Center for Molecular Materials (ICMM), Friedrich-Alexander University of Erlangen-Nürnberg, Nikolaus Fiebiger-Straße 10, 91058 Erlangen, Germany; 3 Department of Neurosurgery, Universitätsklinikum Medical School Erlangen, Friedrich Alexander University of Erlangen – Nürnberg (FAU), Erlangen, Germany; 4 BiMECON Ent, Berlin, Germany

**Keywords:** Cancer, Cell biology, Drug discovery, Neuroscience

## Abstract

Gliomas are primary brain tumors with still poor prognosis for the patients despite a combination of cytoreduction via surgery followed by a radio-chemotherapy. One strategy to find effective treatment is to combine two different compounds in one hybrid molecule via linker to add to or at best potentiate their impact on malignant cells. Here, we report on the effects of a newly synthesized hybrid of sulfasalazine (SAS) and dihydroartemisinin (DHA), called AC254. In previous studies, both SAS and DHA have already proved to have anti-tumor properties themselves and to have sensitizing respectively potentiating effects on other treatments against malignant tumors. We investigated the impact of individual drugs SAS and DHA, their 1:1 combination and a novel SAS-DHA hybrid compound (AC254) on rodent and human glioma cells. In our study SAS alone showed no or only a mild effect on glioma, whereas DHA led to a significant reduction of cell viability in a dose-dependent manner. Next we compared the efficacy of the hybrid AC254 to the combinational treatment of its parent compounds SAS and DHA. The hybrid was highly efficient in combating glioma cells compared to single treatment strategies regarding cell viability and cell death. Interestingly, AC254 showed a remarkable advantage over the combinational treatment with both parent compounds in most used concentrations. In addition to its reduction of tumor cell viability and induction of cell death, the hybrid AC254 displayed changes in cell cycle and reduction of cell migration. Taken together, these results demonstrate that clinically established compounds such as SAS and DHA can be potentiated in their anti-cancer effects by chemical hybridization. Thus, this concept provides the opportunity to devise new effective chemotherapeutic agents.

## Introduction

Primary malignant brain tumors (gliomas) are one of the deadliest neoplasia, which carry poor prognosis for patients despite current aggressive multimodal therapies^1–3^. Human gliomas are highly vascularized^4^ and grow in a space-occupying manner. Hence, the development of new potent agents against gliomas are of high interest. A promising novel approach for generating potent anti-cancer drugs is the hybridization of two or more bioactive compounds. Not only does it keep synthetic effort at a minimum and thus is relatively time and cost saving, but it also can improve upon pharmacological properties of the parent compounds such as increased biological efficacy, reduced undesired side effects, a modified selectivity profile (lower toxicity), better bioavailability, and addition of completely new biological features that were absent in the parent compounds^5–8^. Synthetic hybrids with structures of natural compounds are highly efficient and can be superior to their parent compounds^9–11^. As an example, the artesunic acid-marmycin A hybrid has been reported to be superior to artesunic acid and marmycin A against human osteosarcoma U2OS cells having IC_50_ values 0.23 µM, 1.1 µM, and 14.0 µM, respectively^12^.

Hybrid molecules have significantly increased potency compared to parent compounds. This remarkable behavior could be attributed (***a***) to simultaneous cellular uptake of covalently linked pharmacophores (which is not possible for combination therapy) in a way that the inhibitory kinetics of the two constituents may amplify each other; (***b***) to multivalent binding to protein targets; and (***c***) to increased target binding affinities. Hybrids could also be more stable under metabolic conditions than monomers. Hence, the drug may remain active in the blood stream for a longer time span. Another interesting feature of hybrids—in contrast to parent compounds—is their occasionally reduced toxicity, which can be significant.

In this study we focused on the concept of hybridization, encouraged also by our previous results and experiences with artemisinin-based hybrids^11,13–18^. Artemisinin is a natural anti-malarial compound which was extracted from the Chinese medicinal plant *Artemisia annua* L. (sweet wormwood). The unique structure of artemisinin was elucidated by Youyou Tu in 1972 and her discoveries, concerning a novel therapy against malaria, were awarded by Nobel Prize in Physiology or Medicine in 2015^19^.

**Figure 1:**
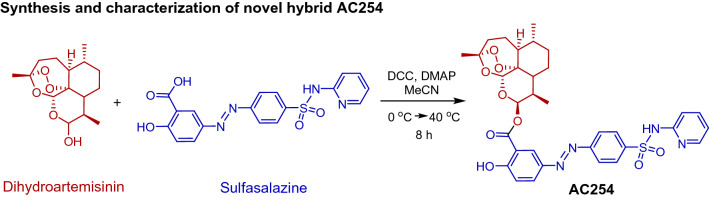
Synthesis of the novel sulfasalazine-dihydroartemisinin hybrid AC254.

There are some artemisinin-derived agents with anti-tumor properties such as artesunate which was found to induce cell death in several types of cancer cells such as pancreatic cancer, T leukemia cells, breast cancer cells and myeloid leukemia cells^20–23^. In this study we focused on the artemisinin derivate dihydroartemisinin (DHA), which is in clinical use as an anti-malarial drug^24^. In the past DHA was already found to hold anti-cancer activity in various types of cancer such as colorectal cancer^25^, lung carcinoma^26^, ovarian cancer^27^, leukemia^28,29^, osteosarcoma^30^, prostate cancer^31^, hepatocellular cancer^32^ and pancreatic cancer^33^ with only mild toxicity toward normal healthy cells^27,31^. The mechanism of its properties consist partly of the induction of apoptosis^26,27,29–33^, cell cycle arrest^26,32,33^, anti-angiogenetic effects^34–36^ and reduction of migration^30^. In recent years DHA has also shown to potentiate the effect of other chemotherapeutic drugs after combining the treatment^26,27^. In search for a compound to combine the artemisinin derivate DHA with, we decided on the anti-inflammatory drug sulfasalazine. Sulfasalazine (SAS) has been developed decades ago as an anti-inflammatory drug and is currently in clinical use for the treatment of chronic inflammatory diseases such as rheumatoid arthritis and ulcerative colitis^37,38^. In various experimental studies SAS has already shown anti-tumor effects on gliomas^39–43^ and other malignant entities such as lymphoma and pancreatic cancer^44,45^. It is especially effective when combining SAS treatment with an established therapy like radiotherapy^43,46^ or chemotherapy^47–50^ in terms of sensitizing malignant cells to treatment and potentiate its effects. Noteworthy, SAS is a pleiotropic drug with various actions including for example induction of cell death^42,51^, inhibition of activation of transcription factor NFĸB^42,45,50,52^ and inhibition of the glutamate antiporter xCT^41,44,45,53^. In particular, the last named mechanism is hypothesized for the anti-cancer action of SAS especially regarding gliomas^41,45^. Furthermore, it shows peritumeral anti-epileptic activity^54^ and is effective in alleviating tumor-induced brain swelling^39^ which are likewise important characteristics for the treatment of brain tumors. Thus, to enhance the pharmacological properties of the selected drugs (DHA and SAS), especially towards brain tumor, we intended to apply the molecular hybridization concept. Herein we report the synthesis of a novel sulfasalazine-dihydroartesiminin hybrid AC254 (Fig. 1), which was investigated for its inhibitory potency against glioma. In this study we performed the proof-of-principle experiments with human glioma cells. However, a great body of glioma research has been investigated in glioma model cells from rats and mice. Thus, we used well established glioma cell line F98 and accompanying control cells in order to make this study comparable to contemporary studies. Notably, AC254 revealed a highly efficient chemotherapeutic effect on gliomas compared to parent compounds and the combinational treatment with both parent compounds in most used concentrations.

## Materials and methods

### Chemicals and general information^11^

Dihydroartemisinin (> 98% (HPLC)) was purchased from TCI Deutschland GmbH, and sulfasalazine (> 98% (HPLC)) was purchased from Sigma Aldrich, Germany. Thin layer chromatography (TLC) was performed on pre-coated aluminum sheets ALUGRAM® SIL G/UV254 (0.2 mm silica gel with fluorescent indicator, MachereyNagel & Co). NMR spectra were recorded at room temperature on a Bruker Avance spectrometer operating at 300 MHz. All chemical shifts are given in the ppm-scale and refer to the nondeuterized proportion of the solvent. ESI mass spectrum was recorded on a Bruker Daltonik micrOTOF II focus TOF MS-spectrometer. Elemental Analysis (C, H, N), carried out with an Elementar vario MICRO cube machine, is within ± 0.40% of the calculated values confirming a purity of > 95%.

### Synthesis and characterization of novel hybrid AC254

In a flame-dried flask, sulfasalazine (100 mg, 0.251 mmol, 1.0 equiv.) and dihydroartemisinin (78.51 mg, 0.276 mmol, 1.1 equiv.) were dissolved in acetonitrile and the solution was cooled down to 0 °C, under nitrogen atmosphere. The coupling reagents DCC (52 mg, 0.251 mmol, 1.0 equiv.) and DMAP (30.66 mg, 0.251 mmol, 1.0 equiv.) were added, respectively. The reaction mixture was warmed up to room temperature in 2 h, following, it was heated up to 40 °C for an additional 6 h. The solvent was removed under reduced pressure and the crude mixture was purified via column chromatography using the solvent mixture hexane and ethylacetate in the ratio 6 to 4, R_f_: 0.24. The pure compound was obtained as yellow powder with 21% yield (ratio C10β/C10α = 75:25). ^1^H NMR (300 MHz, CDCl_3_) *δ* = 8.36 (d, *J* = 2.5 Hz, 1H), 8.34 (d, *J* = 7.1 Hz, 1H), 8.15 (dd, *J* = 9.0, 2.4 Hz, 1H), 8.10 (dd, *J* = 53.7 Hz, 4H), 7.73 (m, 1H), 7.43 (d, *J* = 8.8 Hz, 1H), 7.16 (d, *J* = 8.9 Hz, 1H), 6.85 (t, 1H), 6.54 (d, *J* = 3.3 Hz, 1H), 5.60 (s, 1H), 3.03 (m, 1H), 2.46 (m, 1H), 2.12 (m, 2H), 1.97 (m, 3H), 1.87 (m, 2H), 1.73(m, 2H), 1.44 (s, 3H), 1.38 (m, 2H), 1.12 (m, 2H), 1.02 (d, *J* = 2.4 Hz, 3H), 1.00 (d, *J* = 3.6 Hz, 3H) ppm. ^13^C NMR (125 MHz, CDCl_3_) *δ* = 164.69, 128.03, 123.17, 104.24, 96.56, 94.76, 91.34, 87.92, 81.26, 80.49, 52.86, 52.66, 51.70, 45.60, 44.48, 37.63, 37.52, 36.52, 36.44, 34.91, 34.86, 30.94, 26.22, 26.13, 24.86, 24.82, 24.72, 22.28, 20.51, 20.39, 16.32, 13.33, 12.85. ppm. HRMS (ESI +) *m/z* calculated for C_33_H_35_N_4_O_9_S [M + H]^+^: 663.2137; found: 633.2130. Purity of AC254 was confirmed using elemental analysis, calculated for C_33_H_36_N_4_O_9_S: C: 59.63 H: 5.46 N: 8.43; Found: C: 59.58 H: 5.40 N: 8.89.

### Cell culture glial cell lines

Rodent (F98) and human (U87) cell lines were obtained from ATCC/LGC-2397 (Wesel, Germany). TN22 cells were generated from surgical resection of glioblastoma multiforme patients after informed consent. Primary surgery was guided with with a Siemens Magnetom 1.5 Tesla intraoperative MRI scanner with integrated BrainLab VectorVision neuronavigation. Dissected tumor was classified according to the functional grading according to Friedlein grading: FG A—located in non-eloquent brain areas, FG B—located in the vicinity or in an eloquent brain area^55^. Tissue sample was classified according to the WHO classification of tumor of the CNS. Histopathology was performed by an experienced neuropathologist. TN22 cells were a subclone derived from one female Caucasian patient age 42. TN22 were propagated in Dulbecco’s Modified Eagle Medium (DMEM; Biochrom, Berlin, Germany) supplemented with 10% fetale bovine serum (Biochrom, Berlin, Germany), 1% Penicillin/Streptomycin (Biochrom, Berlin, Germany) and 1% Glutamax (Gibco/Invitrogen, California, USA) cultured under standard humidified conditions (37 °C, 5% CO_2_).From Passage 10 on every 10 passage was freezed and stored at liquid nitrogen over passage 100.

All glioma cells were cultivated in Dulbecco’s Modified Eagle Medium (DMEM; Biochrom, Berlin, Germany) modified with 10% fetale bovine serum (Biochrom, Berlin, Germany), 1% Penicillin/Streptomycin (Biochrom, Berlin, Germany) and 1% Glutamax (Gibco/Invitrogen, California, USA) under standard humidified conditions (37 °C, 5% CO2). After reaching a confluence of approximate 80–90% cells were passaged. For passaging cells were first washed with PBS, then trypsinized, centrifuged (900xT, 5 min, 24 °C) and plated into prepared culture flasks.

### Cell culture primary neurons and astrocytes

Hippocampal neuronal cultures were preserved from the brain of one to four days old Wistar rats (Charles River, USA) by removing the hippocampi from the brain and transferring them into ice cold Hank’s salt solution. After cutting away the dentate gyrus the brain tissue was first trypsinized (5 mg/ml), then triturated mechanically and cultured with MEM medium supplemented with 10% fetale calf serum and 2% B27 Supplement (all from Invitrogen, Taufkirchen). After a short span of time the primary neurons and astrocytes were cultivated in Neurobasal A (Invitrogen, Taufkirchen).

### Cell viability analysis

The cell viability assay was performed using 3(4.5 dimethylthiazol)—2.5 diphenyltetra-zolium (MTT) assay. 3.000 cells/well were plated into 96-well-plate and treated after 2 h. After 72 h MTT solution (Roth, Karlsruhe, Germany) (5 mg/ml) was added and cells were incubated for 4 h. Then MTT solution was sucked away and cells were lysed with 100 μl isopropanol + HCl (110 ml Isopropanol + 330 µl HCl) for 30 min. This described setting was used for the glioma cells F98, U87 and TN22.

Primary neurons and astrocytes were plated into 12-well plates. After 72 h of treatment cells were incubates with MTT solution as described earlier. After 4 h of incubation 200 µl isopropanol + HCL was used for cell lysis. 90 µl of this solution was transferred into 96-well plate for measuring with the microplate reader. The optical density of each well was measured using the microplate readaer Tecan Infinite F50 (Crailsheim, Germany) set to 550 nm using i-control software. The viability of the different treatments is expressed as the percentage of cells without any treatment.

### Cell death assay and apoptosis analysis

100,000 or 200,000 cells/well were plated into 6-well-plates and again treated after 2 h with 1 µM. After 24 or 72 h medium and cells were collected, washed with PBS and stained with 0.1% Annexin V and 0.1% 7-ADD (Biolegend, San Diego, USA). Results were obtained by Flow Cytometer BD FACSCanto II (BD Bioscience, Heidelberg, Germany) and analyzed with WinList 3D 8.0 software.

### Phalloidin staining procedure

For the staining of cytoskeleton 50,000 cells were seeded/well on glass cover slides in a 12-well plate. Treatment followed after 5 h. 24 h after treatment cells were fixated with PFA 4% and stained with Phalloidin 488 (1:50) and Hoechst (1:10 000). Pictures were taken by Axio Observer with Zen Software (Zeiss, Oberkochen, Germany).

### Cell migration assay

100,000 cells/well were plated into 12-well plate. A sratch was made with a sterile pipette tip right through the layer of cells. Images were taken (0 h) and cells were treated with different concentrations (0.5 µM and 5 µM) of the compounds. Pictures were taken by Olympus × 71 and cell-F-Software (Olympus, Tokyo, Japan) at various times. To analyze the data the distance between cells was measured using Image J software.

### Cell cycle analysis

For cell cycle analysis 100,000 cells/well respectively 200,000 cells were seeded in 6-well dishes. After 2 h cells were treated with 1 µM of the different compounds and their combination. After 24 h respectively 72 h cells and the media supernatant were collected and washed with PBS. Then cells were fixated with cold 70% ethanol for 30 min at 4 °C. After another wash step with PBS RNAse A (100 μg/ml) and 7AAD solved in PBS were added to the cells for 30 min at room temperature. Cell cycle analyses were performed by Flow Cytometer BD FACSCanto II (BD Bioscience, Heidelberg, Germany). Analysis were made with Flowing Software 2 and WinList 3D 8.0.

### Hoechst assay

100,000 cells/well were plated into 6-well-plates, after 2 h cells were treated. After 72 h of treatment medium was removed and cells washed with PBS. Cells were trypzinized and pooled with the removed medium. Cells were again washed with PBS and fixated with 4% PFA for 10 min. After another wash step cells were incubated with Hoechst (2 µg/ml) for 30 min. Then cells were moved to microscope slide. Pictures were taken with 40 times enlargement by an Axio Observer with the Zen Software (Zeiss, Oberkochen, Germany).

### Statistical analysis

Quantitative data from experiments were obtained as stated in the figure legend. Analysis was performed using one- or two-way ANOVA (α = 0.05) and Scheffé’s method (MS Excel). The level of significance was set at *p* < 0.05. Data from all experiments were obtained from at least three independent experiments. Error bars represent ± SEM.

### Ethical approval

All methods were carried out in accordance with relevant guidelines and regulations. Animal killing was performed in accordance with the German Protection of Animals Act 14 paragraph one and three. The announcement of rat and mouse killing was approved by the designee for animal protection of the University of Erlangen-Nuremberg (TS-7/12). The study was carried out in compliance with the ARRIVE guidelines. Studies with human tissue were conducted in compliance with the Helsinki Declaration and approved by the Ethics Committee of the Friedrich Alexander University of Erlangen-Nuremberg. All patients gave written informed consent to donate tissue for use in the study.

## Results

### The impact of hybrid AC254 on cell morphology

**Figure 2:**
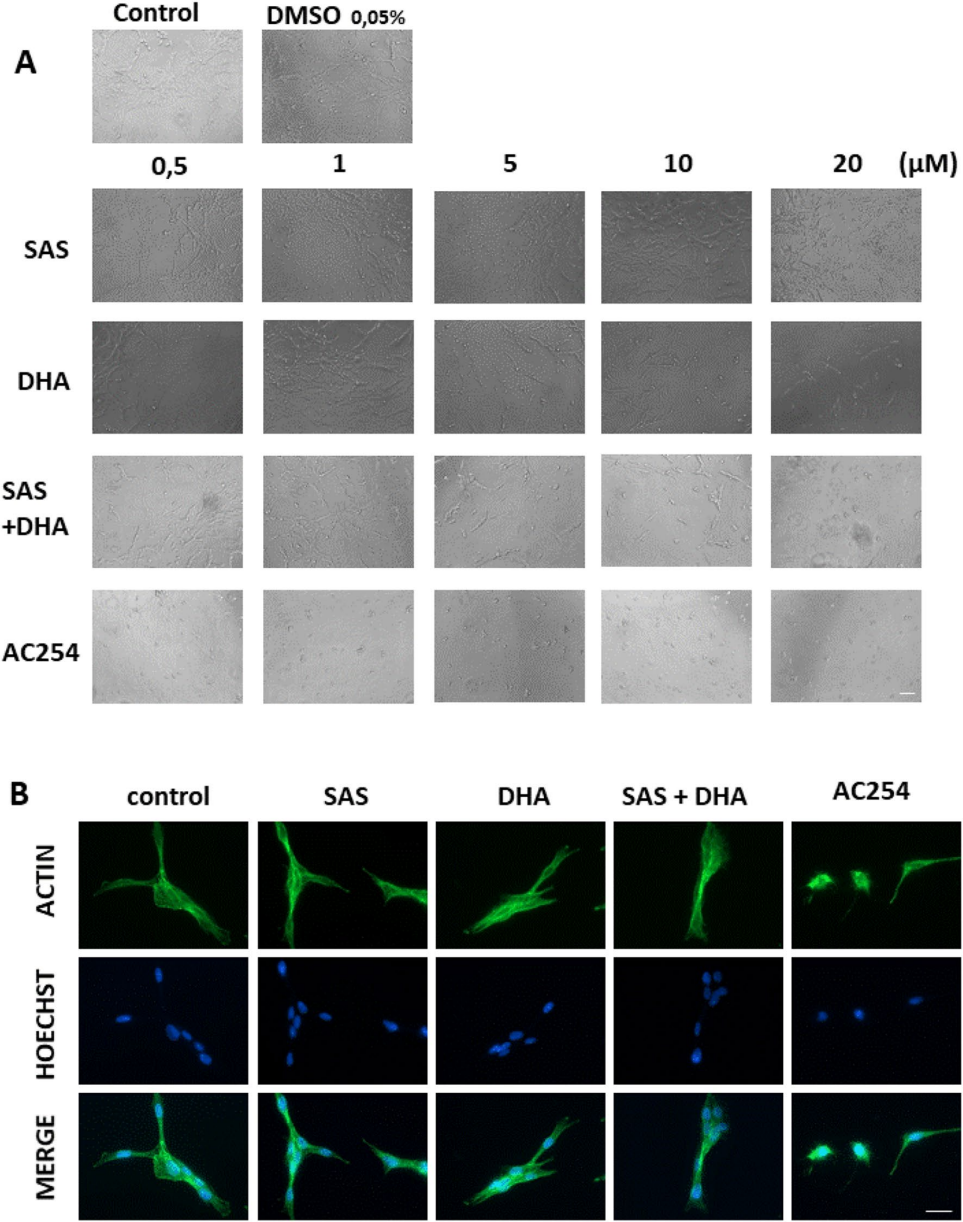
Morphologic profiling of Sulfasalazine, DHA and the novel hybrid compound AC254 on rodent glioma cells. (**A**) Rat glioma cells (F98) were treated with a range of concentrations of SAS, DHA, AC254, SAS and solvent DMSO combined with DHA. Cell morphology was examined after 72 h with light microscopy. Olympus × 71 with cell F-Software (Olympus). Scale bar, 200 µM. (**B**) F98 were treated with 0.5 µM of SAS, DHA, AC254 and SAS combined with. Cells were fixated and stained with actin marker Phalloidin 488 and DNA marker Hoechst after 24 h of treatment. Axio Observer with Zen Software (Zeiss). Scale bar: 20 µm.

The first step was to examine the effect of the parent compounds SAS and DHA, their combination and the hybrid on glioma cell morphology using different concentrations. The examination took place after 72 h of treatment (Fig. 2).

The confluence of glioma cells treated with SAS was similar to the confluence visible in the control even when using higher concentrations (Fig. 2A). However glioma cells treated with DHA behaved differently. In low concentrations there was no effect visible (0.5 µM, 1 µM) (Fig. 2A). The cells looked equal to the control in shape and confluence. Whereas using DHA in higher concentration an increasing impact on cell morphology was visible. There were less cells per area, the cells had less branches and some of them even had a round cell body (5 µM, 10 µM, 20 µM) (Fig. 2A). When treating gliomas with a 1:1 mixture of SAS and DHA they respond similar to cells treated with sole DHA (Fig. 2A). In low concentration cells did not seem to react to the treatment, only when increasing the concentration the cells changed their appearance. Interestingly using light microscopy detected a considerable difference between cells treated with the hybrid AC254 and cells under controlled conditions already in low concentrations (0.5 µM). The cells retracted their membrane extensions, rounded up and had no polygonal shape at all. This effect was displayed in all used concentrations in the same extent (Fig. 2A). To further investigate the morphological change we had a closer look on the glioma cytoskeleton. Therefore cells were imaged for actin filaments and DNA after 24 h of treatment with 0.5 µM (Fig. 2B). We found that both SAS and DHA did not alter significantly cortical and cytoskeletal actin polymerization (Fig. 2B). The same was true for the SAS and DHA mixture in equal parts. In contrast, the hybrid compound AC254 led to massive actin polymerization in the cytoskeleton (Fig. 2B).

Summarized these findings indicate that the hybrid AC254 is most effective in changing the shape of glioma cells compared to the single treatment of its parent compounds SAS and DHA and there 1:1 combination. Furthermore it shows that linking molecules together can create a new compound with new properties.

### The impact of AC254 compared to its parent compounds on cell viability

**Figure 3:**
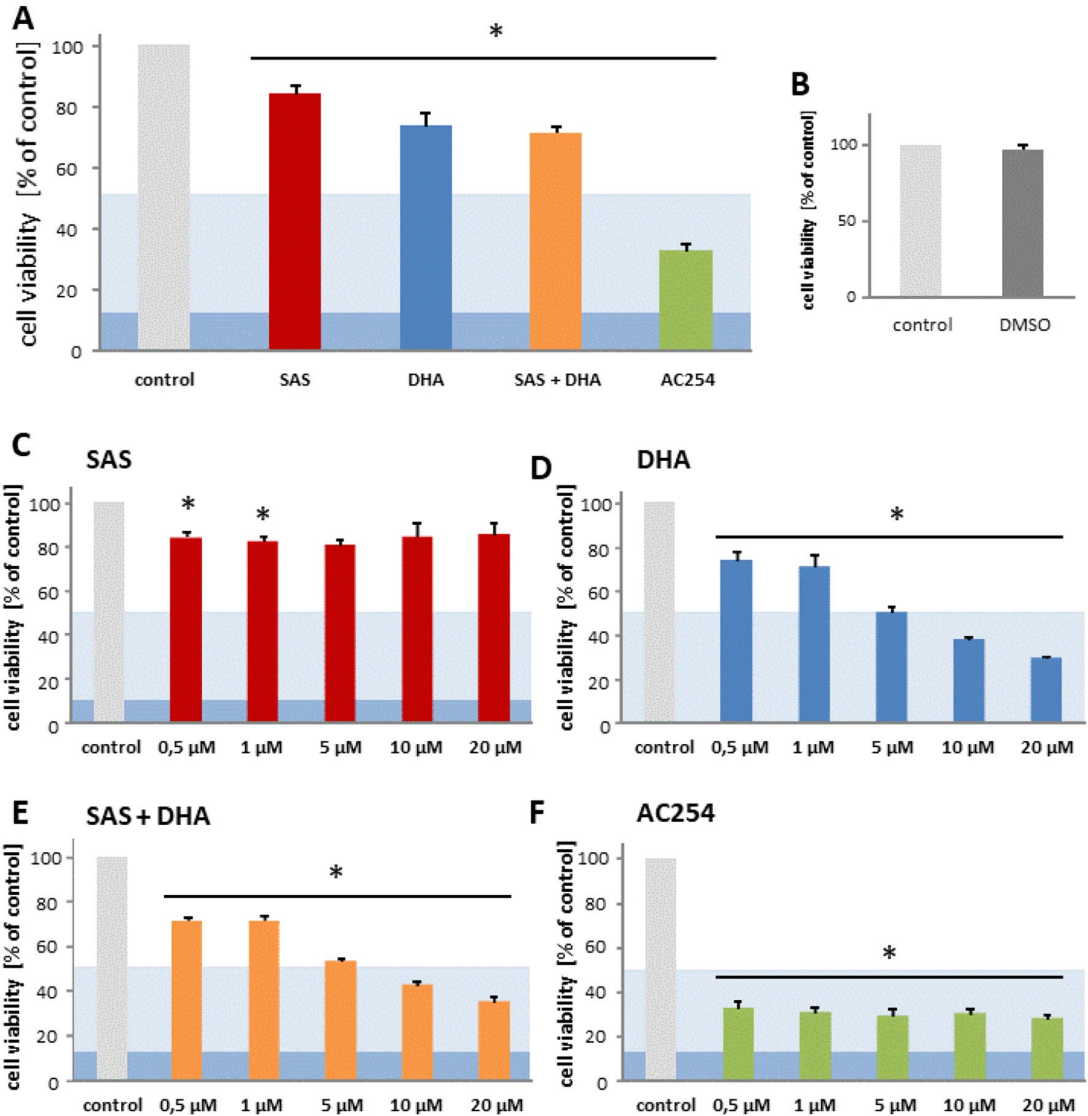
Cytotoxic profiling of Sulfasalazine, DHA and the novel sulfasalazine-dihydroartemisinin hybrid AC254 on rodent glioma cells. (**A**) Cells were treated with 0.5 µM of SAS, DHA, AC254 and the combination of SAS and DHA. Cell viability was measured after 72 h. n ≥ 4. * indicates significance to control (*p* < 0.05). The light blue marking indicates the value of IC50 and the dark blue one stands for IC 90 value. This is passable for the following figures. (**B**) Cells were treated with 0.05% DMSO which represents the maximum concentration of DMSO achieved at the treatment with 20 µM at the combinational treatment of SAS and DHA. At all other treatments the concentration of DMSO was less. Measurements were made after 72 h. (**C**–**F**) Cells were treated the same way as in A with different concentrations of the compounds, SAS (**C**), DHA (**D**), SAS combined with DHA (**E**) and the hybrid AC254 (**F**). n ≥ 4. * indicates significance to control (*p* < 0.05).

Next we examined the impact of SAS, DHA, their combination and the covalent bound hybrid of SAS and DHA on rodent glioma cells. Hence, the F98 glioma cells were treated with various concentrations of sole SAS, sole DHA, their 1:1 mixture and the hybrid termed AC254. We investigated with this approach the impact of SAS and its hybrid molecule on primary brain tumors, in particular gliomas (Fig. 3).

For this we analyzed the consequences of the treatment with a wide range of concentrations on cell viability after 72 h of treatment. First looking at the parent compounds our experiments showed no or rather a slight difference when treating cells with SAS compared to control (Fig. 3A,C). Comparing the various concentrations of SAS with each other showed no significant difference in efficiency (Fig. 3C). These results confirm previous studies with SAS on glioma cells which found an effective concentration of SAS for reducing cell viability at 200 µM^39^. The other parent substance DHA however decreased cell viability already significant in a low concentration (0.5 µM) (Fig. 3A, D). This effect is increasing in a dose-dependent manner. At the highest concentration we used (20 µM) in our study glioma cell viability was reduced to a level of approximately 30% when treating with DHA (Fig. 3D).

To exclude the possibility that the solvent DMSO used for solving the compounds had any effects on our results, we performed the cell viability assay and the microscopy also with the highest concentration of DMSO (0.05%). We used this concentration only for the combinational treatment of SAS and DHA at 20 µM. Noteworthy, DMSO had no significant effect on cell viability (Figs. 2A, 3B). All the other treatments facilitated much lower DMSO concentrations.

Next, we focused on the impact of the 1:1 combination of SAS and DHA. We found that the mixture of SAS and DHA had similar impact as single treatment of DHA (Fig. 3A,D,E). The combination of the parent compounds reduced cell viability significantly as well as DHA in a dose dependent manner (Fig. 3E). Comparing the 1:1 mixture of SAS and DHA to sole use of DHA no significant advantage of one another based on our results of cell metabolism could be found. Cells treated with the covalently bound hybrid of SAS and DHA termed AC254 showed a strong reduction of cell viability (Fig. 3A,F). The hybrids impact was already obvious at low concentrations (0.5 µM) and could not be increased by increasing the compound’s quantity (Fig. 3F). It stays on a level of cell viability comparable to the effect of DHA when utilized on a very high concentration (20 µM) (Fig. 3D,F). The impact of DHA mixed with SAS is significantly potentiated by AC254 in lower concentrations (0.5 µM, 1 µM, 5 µM) (Fig. 3E,F).

Taken together these results match to the previous findings in cell morphology and emphasize that linking SAS and DHA in a covalent manner can change the biological impact of these compounds on gliomas heavily.

### AC254 is a potent gliomatoxic compound

**Figure 4:**
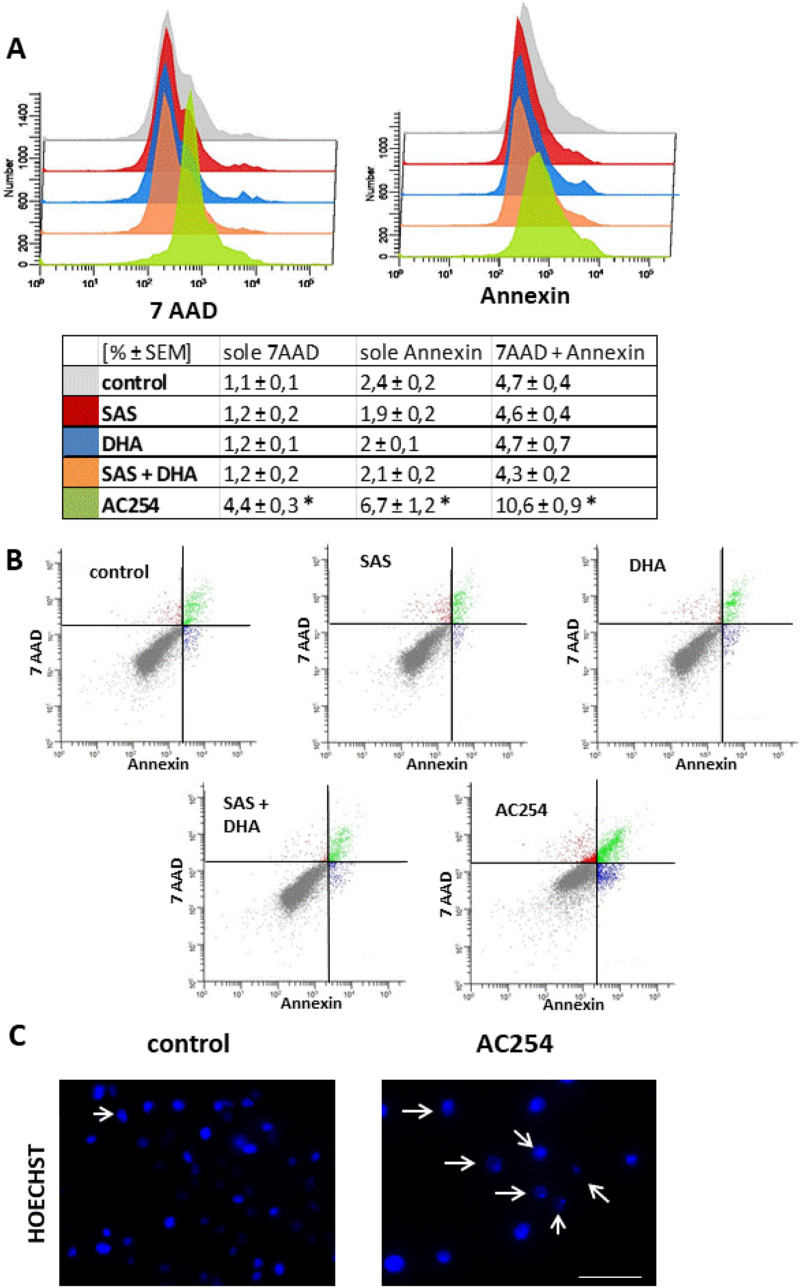
Cell death analysis after following application of compounds. (**A**, **B**) F98 rodent glioma cells (100.000 cells/well; 6 well) were treated with 1 µM of the compounds. Cell death analysis was performed after 72 h with 7 AAD and annexin V. n ≥ 5. *indicates significance to control (*p* < 0.05). (**C**) F98 were treated the same was as in (**A**, **B**). After 72 h F98 were collected and stained for the DNA marker Hoechst. C pictures were taken with Axio Observer with Zen Software (Zeiss). Scale bar, 50 µm.

The question remains which mechanisms contribute to the reduction in cell viability and changes in cell morphology. To investigate whether our so far detected effects of the new compound AC254 can be attributed to cell death or might have other reasons we performed a flow cytometry with the marker 7-Amino-actinomycin (7-AAD) and annexin V. Therefore, cells were treated with 1 µM of the compounds for 72 h (Fig. 4).

The results revealed that the non-treated cells as well as the cells treated with SAS, DHA and their combination were approximately 92% viable (Fig. 4A,B). There was no significant difference between the composition of 7AAD and annexin V positive cells comparing the treatment with SAS, DHA and their 1:1 mixture to control (Fig. 4A,B). Whereas after the treatment with AC254 only about 80% of the cells were still alive (Fig. 4A,B). Cells treated with AC254 showed a significantly higher amount of 7AAD and annexin V positive cells compared to control as well as to all other treatments. The quantity of 7AAD-positive and annexin V-negative cells was around 4 times more compared to control (Fig. 4A,B). The amount of sole annexin V and both positive cells was about 2–3 times higher compared to the other groups (Fig. 4A,B). These findings demonstrate that AC254 induces apoptosis as well as other ways of cell death. However, apoptosis seems to be the major part of the induced cell death. To verify these results we performed a morphological apoptosis assay with Hoechst staining (Fig. 4C). Here, we found that treating glioma cells with AC254 led to nuclear bloating and nuclear fragmentation (Fig. 4C). In summary these experiments underline that the reduction in cell viability and the changes in cell morphology can be attributed to the induction of cell death induced by AC254. Thus, AC254 can be considered a novel compound with cytotoxic potential to combat gliomas.

### AC254’s effect on cell cycle

**Figure 5:**
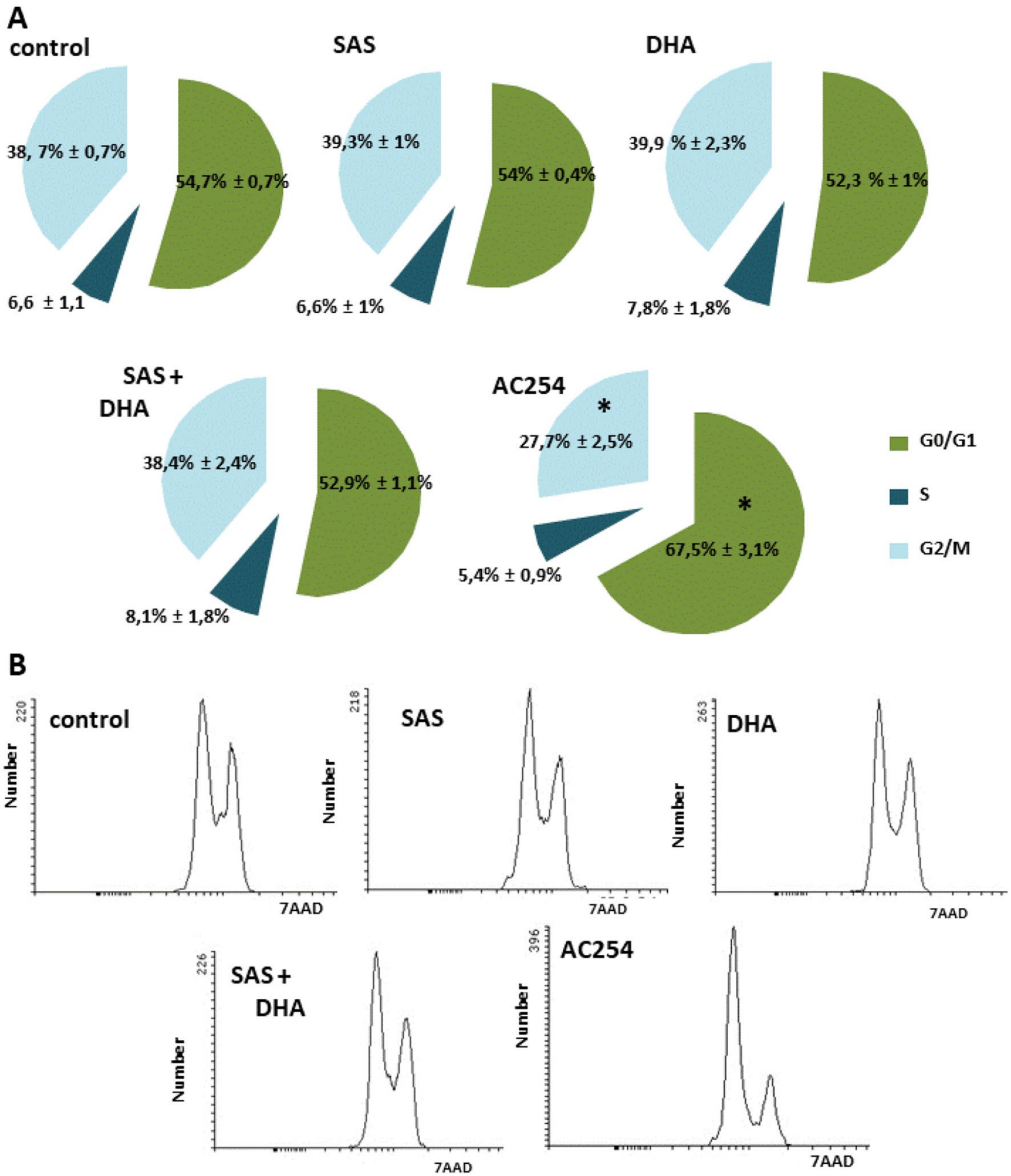
Cell cycle analysis after 72 h of treatment. (**A**, **B**) F98 (100,000 cells/well; 6 well) were treated with 1 µM of SAS, DHA, AC254 and the combination of SAS and DHA. Cell cycle analysis was performed after 72 h after fixation and staining with 7AAD. N ≥ 5. * indicates significance to control (*p* < 0.05).

Furthermore, we performed cell cycle analysis with the rodent cell line F98. For this cells were treated 2 h after plating for duration of 72 h, fixated and stained for 7AAD (Fig. 5). Under controlled conditions about 55% of the cells were in G0- and G1-phase, around 7% were in S-phase and another 39% were located in the G2- and M-phase (Fig. 5A). Between cells treated with SAS, DHA, their 1:1 mixture and non-treated cells there was no significant difference in cell cycle visible (Fig. 5A,B). However, the treatment with AC254 showed a significant change in cell cycle. There was a significant increase of cells in G0- and G1-phase (Fig. 5A) which indicates that AC254 stops cells from being part of cell division. Another hint at this was the significant reduction of cells in G2- and M-phase after being treated with AC254 (Fig. 5A,B). Taken together, these findings show that the hybrid AC254 does not only lead to cell death but also slows down cell replication while its parent compounds or combination do not display such effects.

### Time of duration matters

**Figure 6:**
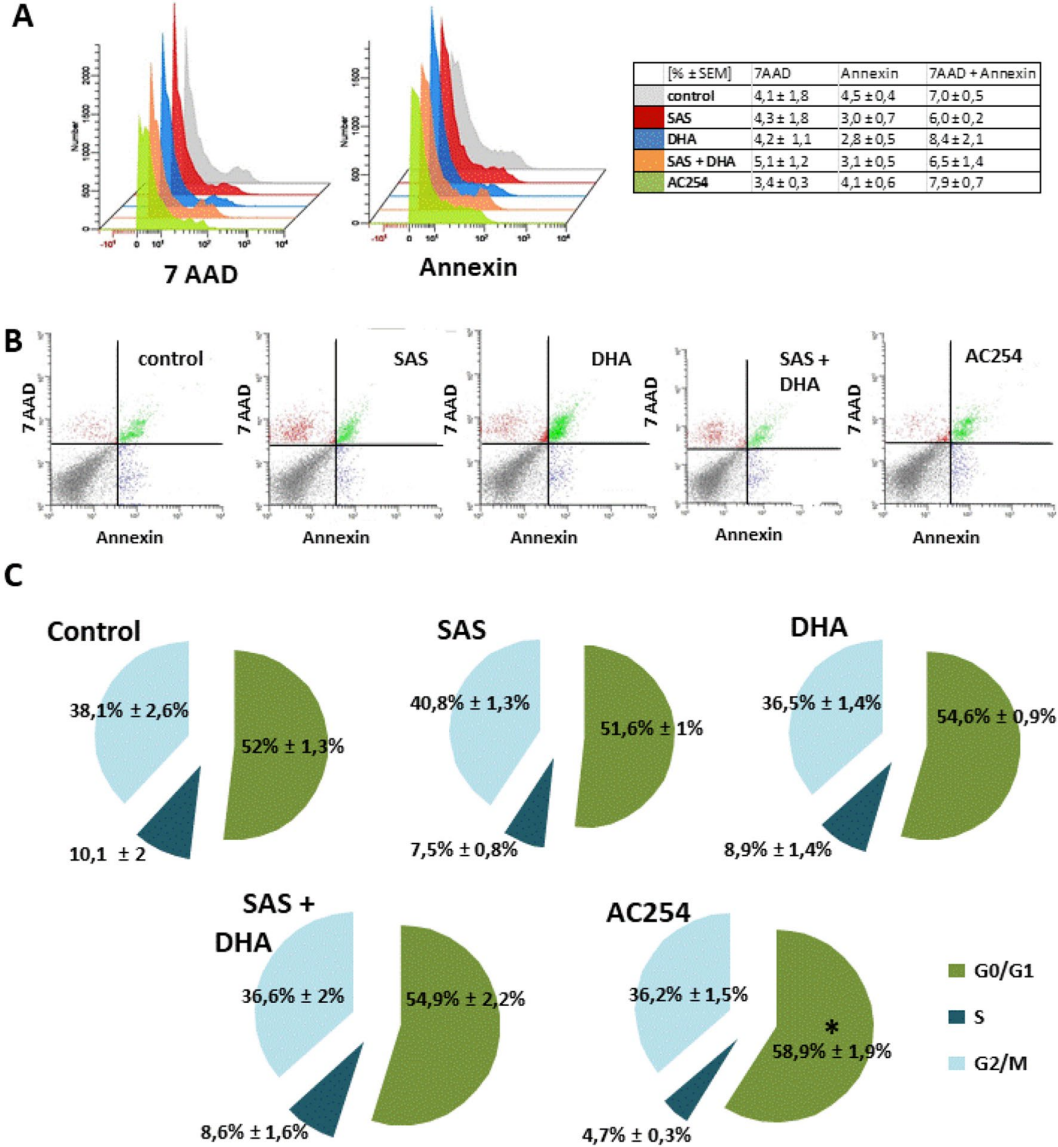
Cell death analysis and cell cycle after 24 h of treatment. (**A**, **B**) Rodent cells (200.000 cells/well; 6 well) were treated with 1 µM of the compounds. Cell death analysis was performed after 24 h with 7 AAD and Annexin V. n = 3. (**C**) F98 were treated in the same way as in (**A**, **B**). Cell cycle analysis were performed after 24 h after fixation and staining with 7AAD. n ≥ 3.

Because we detected morphological changes after treatment with AC254 already after 24 h we wanted to investigate whether time matters for the gliomatoxic effect. Hence cell death and cell cycle analysis were repeated after 24 h with a 1 µM treatment on rodent cells (Fig. 6). The results showed no increase in early apoptosis and cell death in general for all treatment groups compared to cells under controlled conditions (Fig. 6A,B). We further investigated the cell cycle after 24 h of treatment. As expected SAS, DHA and their combination did not change cell cycle compared to control (Fig. 6C). Additionally also AC254 showed no significant modification in cell phases (Fig. 6C). All in all these experiments indicate that the mechanisms leading to cell death take more than 24 h in the case of the compound AC254 to unfold its gliomatoxic effect.

### AC254 acts chemotherapeutic on human glioma cells

**Figure 7:**
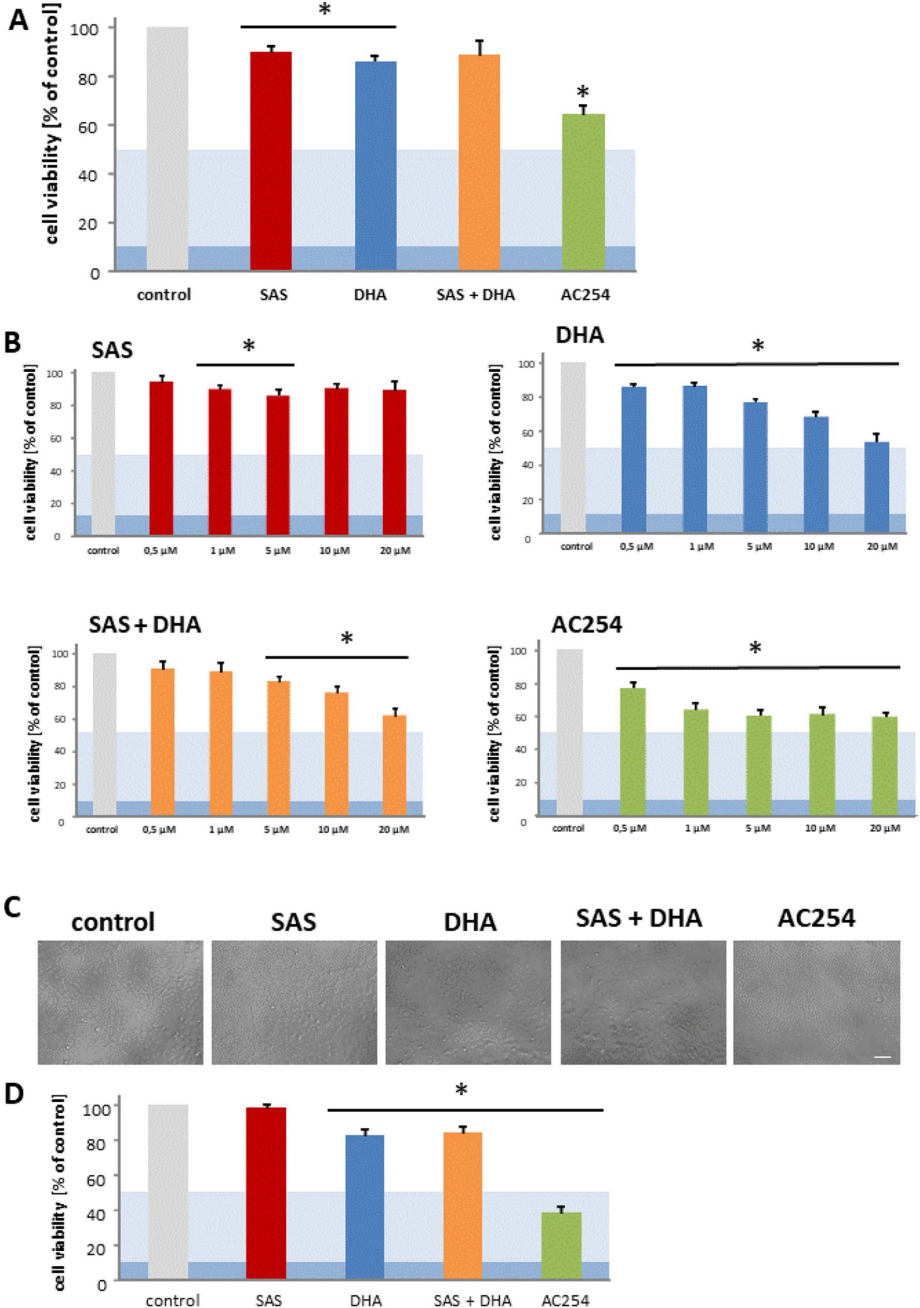
Effects on human cell viability (U87, TN22). (**A**) Cells (U87) were treated with 1 µM of the compounds. Cell viability was measured after 72 h. n = 4. * indicates significance to control (*p* < 0.05). (**B**) U87 were treated with various concentrations of the compounds. Cell viability was measured the same way as in A. n = 4. * indicates significance to control (*p* < 0.05). (**C**) TN22 were treated with 5 µM of the compounds. Cell morphology was examined after 72 h with light microscopy. Olympus × 71 with cell F-Software (Olympus). Scale bar, 200 µm. (**D**) TN22 were treated with 5 µM of the compounds, cell viability was measured after 72 h. n = 4. * indicates significance to control (*p* < 0.05).

**Figure 8:**
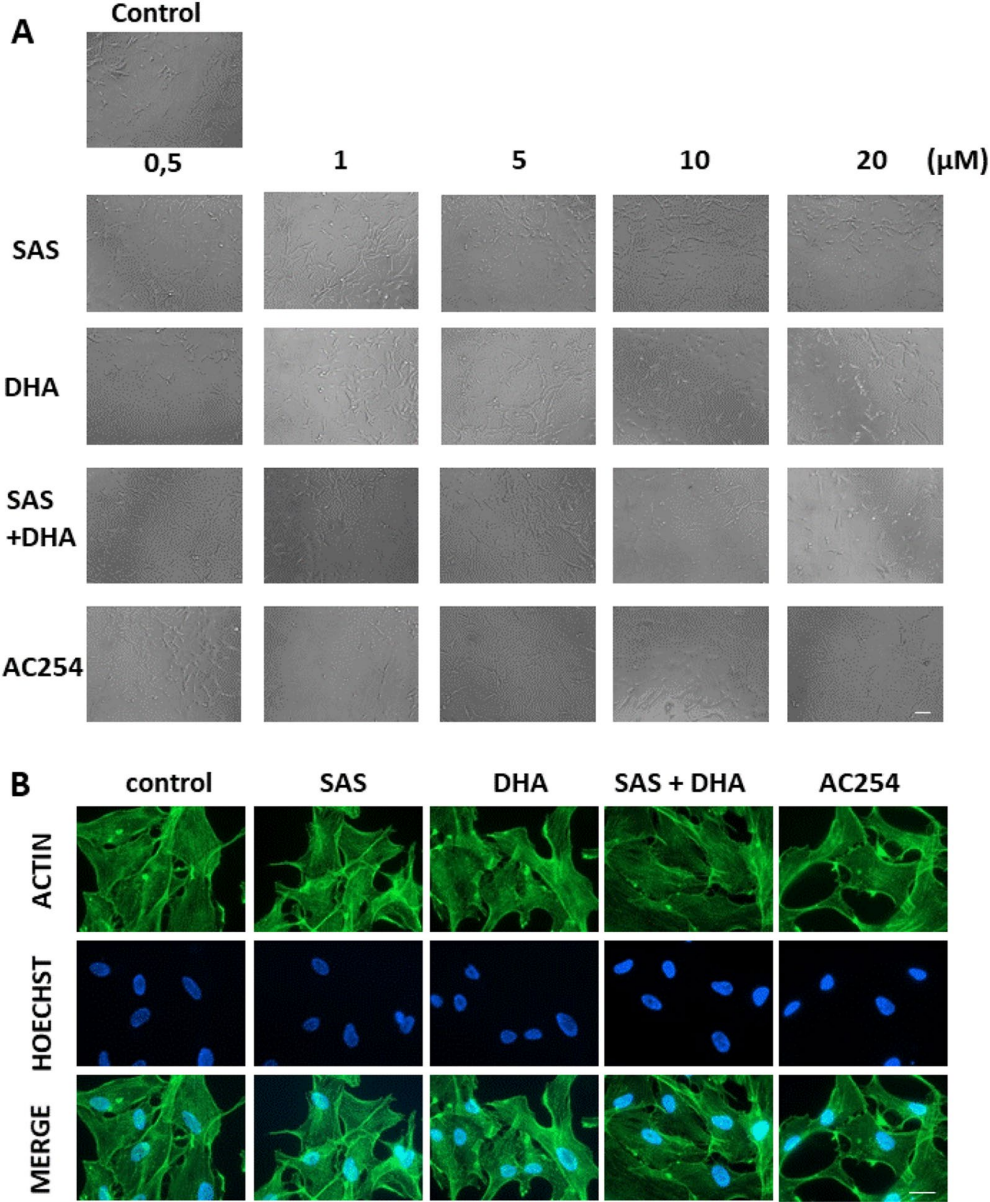
Impact on human cell morphology. (**A**) Human cells (U87) were treated with a variation of concentrations of SAS, DHA, their covalently bound hybrid (AC254) and their 1:1 mixture. Cell morphology was examined after 72 h with light microscopy. Olympus × 71 with cell F-Software (Olympus). Scale bar, 200 µM. (**B**) Cells (U87) were treated with 5 µM of SAS, DHA, AC254 and SAS combined with. Cells were fixated and stained with actin marker Phalloidin 488 and DNA marker Hoechst after 24 h of treatment. Axio Observer with Zen Software (Zeiss). Scale bars represents 20 µm.

Next, we examined the impact of our compounds on human glioma cells. Therefore, we treated U87 with SAS, DHA, AC254 and the combination of the single compounds with different concentrations (0.5 µM, 1 µM, 5 µM, 10 µM and 20 µM) for 72 h (Fig. 7). The first step was to view cell viability. SAS showed no significant impact on human glioma cell line U87 in all used concentrations (maximum 20 µM) (Fig. 7A, B). Whereas DHA reduced cell viability in a dose-dependent manner beginning with a slight non statistic reduction of cell viability in low concentrations (0.5 µM, 1 µM) and a significant reduction in higher concentrations (5 µM, 10 µM, 20 µM) (Fig. 7A,B). When treating cells with a combination of SAS and DHA, the cells again showed almost the same level of reduction in cell viability as cells treated with sole DHA. We could not show any significant difference between these two treatments regarding all used concentrations (Fig. 7A,B). Raising the concentration increased the effect of the treatment. Noteworthy, the hybrid AC254 was most effective in reducing the cell viability especially in low concentration (Fig. 7A, B). At the concentration of 1 µM and 5 µM AC254 reduced cell viability significantly compared to the impact of all other treatment groups (Fig. 7A, B). In higher concentrations (10 µM, 20 µM) there was no significant difference visible comparing AC254 treatment to DHA and its combination with SAS (Fig. 7B). Whereas DHA operated in a strong dose-dependent manner, AC254 did only amplify its impact on human glioma cells mildly when increasing its concentration (Fig. 7B). We further had a look at cell confluence after treating the cells with all compounds. Despite the effect in cell viability we could not detect a difference in confluence comparing the treatments with the control (Fig. 8A). Likewise, staining the cells for actin filaments and nuclei did not show any effects of the compounds regarding actin polymerization at 24 h and at 5 µM (Fig. 8B).

To strengthen these results we examined the impact of all compounds on another human glioma cells line (TN22) for a single dose (5 µM). We checked cell viability and morphology (Fig. 7C,D). Cells treated with SAS showed no significant impact compared to cells under controlled conditions regarding cell viability whereas the treatment with sole DHA and the combinational treatment with SAS led to a significant reduction in cell viability at the comparable level (Fig. 7D). Yet again the hybrid AC254 was most effective in reducing cell viability in a significant manner comparing its impact to those of the other treatments (Fig. 7B,D). Interestingly using light microscopy there was a difference in cell confluence visible between cells treated with AC254 and cell under controlled conditions (Fig. 7C). In summary, these results confirm our former findings on rodent glioma cells though we detected a difference in efficiency between the different cell lines. SAS does not seem to be a gliomatoxic agent in low dose, whereas DHA and especially the covalently bound hybrid of SAS and DHA seems to be highly potent at killing glioma cells.

### Impact on cell migration and glioma invasive growth

**Figure 9:**
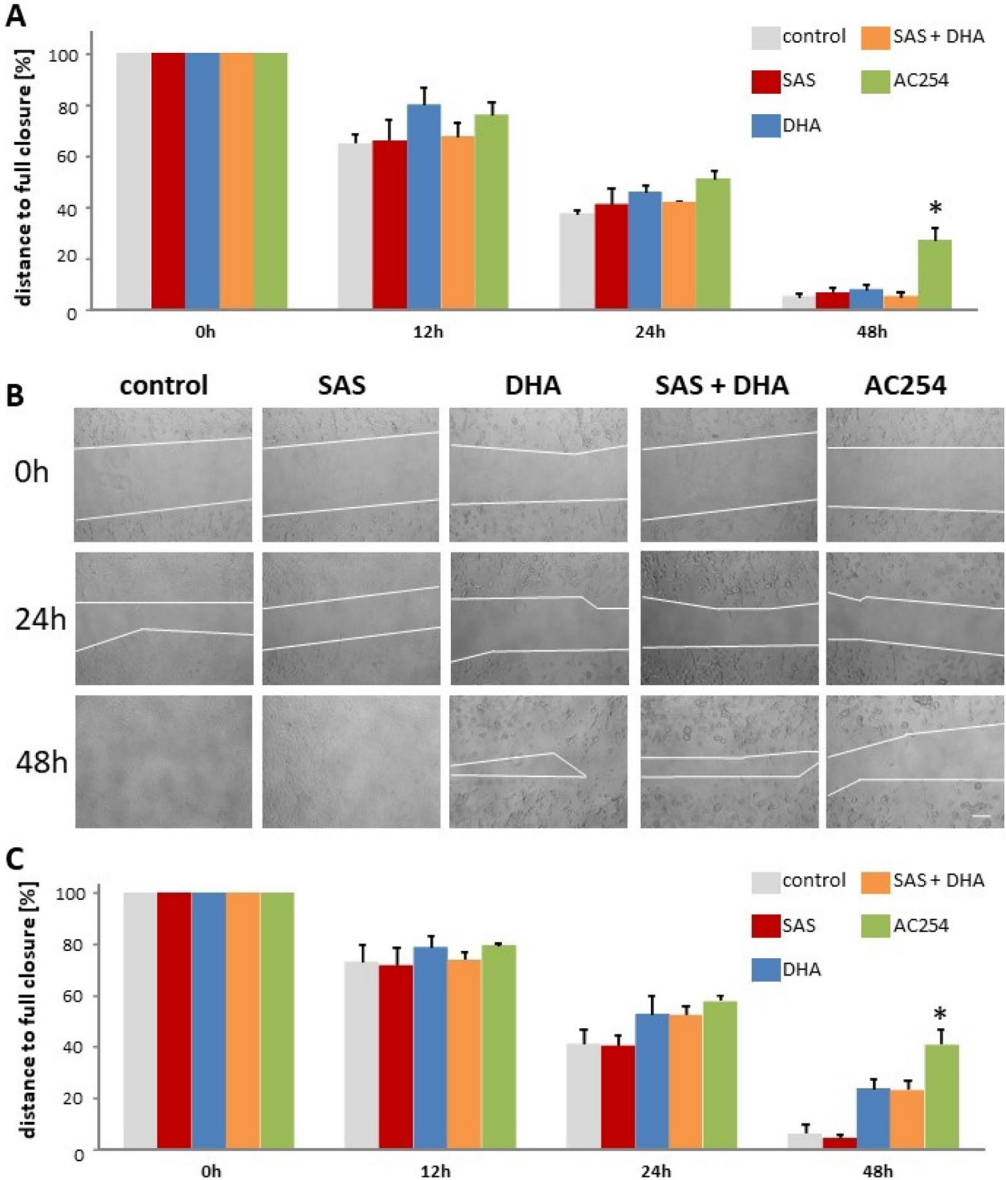
Impact of the compounds on cell migration. (**A**, **B**, **C**) A scratch was made into plated cells (F98) after 36 h. Cells were then treated with 0.5 µM (**A**) and 5 µM (**B**, **C**) of the compounds. Representative images were taken (Olympus × 71 with cell F-Software (Olympus)). (**B**), quantified and related to the time the scratch was made (0 h) (**A**, **C**). n = 3. * indicates significance to control (*p* < 0.05).

Another important property especially for anti-glioma drugs is to suppress cell migration. To investigate this we plated rodent cells into a 12-well plate, after 36 h a scratch was made, afterwards cell were treated with 0.5 µM (Fig. 9A) and 5 µM (Fig. 9B,C) of the compounds and monitored at various times. 12 h after the treatment the gap between the cells in all treatment groups and both concentrations was similar to each other (Fig. 9A–C). It took a minimum 24 h for the hybrid AC254 to achieve a minor non-significant advantage in terms of a larger gap over the control and the other treatments in both concentrations (Fig. 9A–C). After 48 h, the treatment with AC254 showed significantly more impact on the cells than the control and the different treatments, the gap’s size was significantly larger than the one of the control’s. Instead, SAS, DHA and their combinational treatment seemed to have no significant effect when using the smaller concentration of 0.5 µM (Fig. 9A). While AC254 was effective in both concentrations, DHA and the mixture of DHA and SAS decreased the gap closure only in higher concentration in a non-significant way (5 µM) (Fig. 9A–C).

In summary, the new hybrid inhibits the wound’s closure which might correlate with cell migration. In this setting it cannot be certainly differentiated between a gap closure because of cell migration or cell multiplication. Besides that the effect of AC254 showed in this assay can be an important trait for anti-cancer drugs.

### Primary neurons and astrocytes

**Figure 10:**
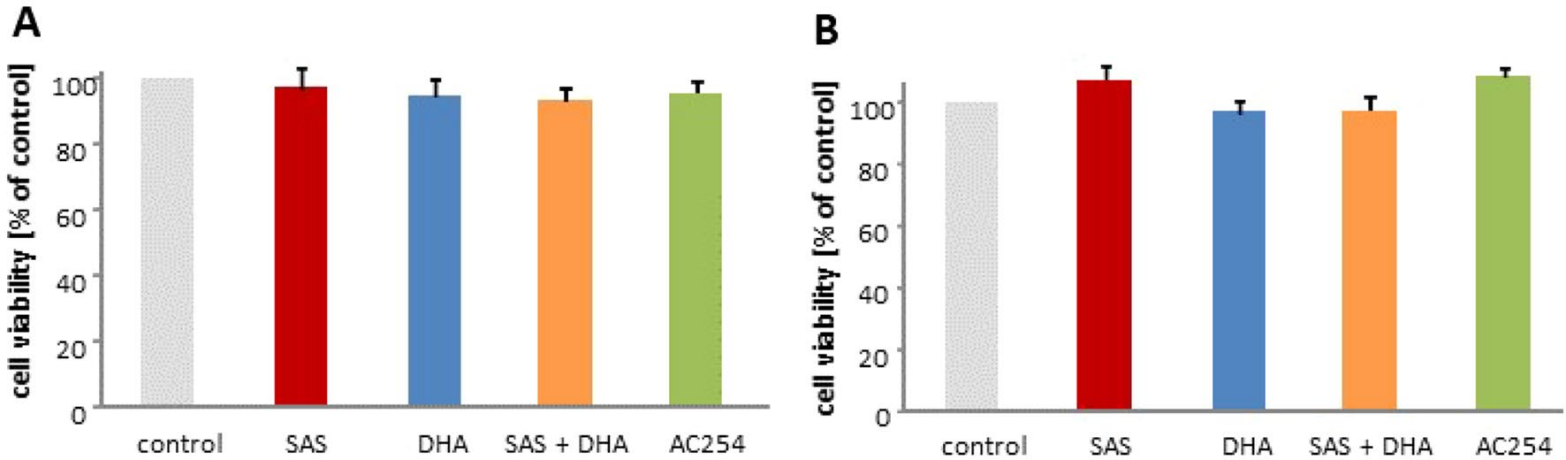
Neurotoxicity an gliomatoxicity profiling. (**A**,**B**) Cells were treated with 1 µM (**A**) and 5 µM (**B**) of the compounds. Cell viability assay was performed after 72 h. n ≥ 6. * indicates significance to control (*p* < 0.05).

The next step was to investigate whether the compounds show any effect on healthy brain cells. Therefore, primary neurons and astrocytes were isolated from rodent brain and treated with two different concentrations of all compounds (Fig. 10).

We performed a cell viability assay for 1 µM and 5 µM after 72 h. There we found no significant difference in cell viability comparing the different treatments with the cell viability under controlled conditions (Fig. 10A,B). This suggests a minor impact on healthy brain.

## Discussion

In general, the aim of chemotherapy is killing malignant cells while not harming the surrounding non-transformed cells. In neurooncology, glioma cells represent the therapeutic targets and drugs should be facilitated at concentrations where normal healthy tissue (e.g. neurons, astrocytes) has the chance to survive. This cytotoxic strategy therefore depends on compounds which fulfill the requirements to be specifically toxic to cancer cells. Another strategy is to combine two effective anti-tumor agents in order to keep the necessary amount of chemotherapeutic substance as low as possible. In this study we combined these two methods especially. Two well-known compounds with a long clinical use were bound together covalently. One of the promising molecule is dihydroartemisinin (DHA). DHA is a derivative of the natural antimalarial compound artemisinin. Additionally to its anti-malaria effects^24^ DHA has already proved being an effective agent against various types of malignant tumors such as colorectal cancer^25^, lung carcinoma^26^, ovarian cancer^27^, leukemia^28,29^, osteosarcoma^30^, prostate cancer^31^, hepatocellular cancer^32^ and pancreatic cancer^33^. There are reported studies in which DHA treatment was combined with other chemotherapeutic drugs, they showed that DHA can potentiate the effect of single drug treatment^26,27^. The mechanism of its properties is not fully understood yet but probably consists of the induction of apoptosis^26,27,29–33^, cell cycle arrest^26,32,33^, anti-angiogenetic effects^34–36^ and reduction of migration^30^. As good sister molecule appeared sulfasalazine (SAS). SAS as well as DHA has a long clinical history and is commonly used for inflammatory diseases such as ulcerative colitis and rheumatic arthritis^37,38^. In the past it has already shown some important properties especially when dealing with brain tumors. SAS inhibits the glutamate transporter xCT which is an essential oncogene in brain tumors and other tumor entities^41,44,53^, it shows peritumoral anti-epileptic activity^54^ and is effective in alleviating tumor-induced brain swelling^39^. To get reliable data we investigated the impact of SAS and DHA on various glioma cells as single compounds, in combination with a ratio of 1:1 mixture of both single drugs and as a chemical linked hybrid of SAS and DHA termed AC254. Our experiments showed that SAS has no or only a minor effect on glioma cells regarding cell morphology, cell viability, cell death, cell cycle and cell migration in the concentrations used by us. These findings match previous results on glioma cells in which higher concentrations of SAS were needed to show any effects^39^. In turn, we found DHA to reduce cell viability significantly already in low concentration.

The combinational treatment with SAS and DHA showed no significant improvement to sole treatment with DHA in all our settings which leads to the conclusion that the combination of SAS and DHA has no benefit to sole DHA therapy. Delightfully linking SAS and DHA chemically together can potentiate the effect of DHA treatment significantly. The hybrid of SAS and DHA (AC254) led to strong reduction of cell viability which can be attributed to cell death especially apoptosis and an arrest in cell cycle. Another trait of AC254 is the inhibition of cell migration which is a very important property to combat malignant tumors especially gliomas which tend to disseminate throughout the brain.

Summarizing, this study shows that it is possible to amplify an anti-glioma drug’s properties by hybridizing it with another drug or biologically active compound, and illustrates the high potential of the hybridization concept as an alternative drug‐discovery approach.

## Supplementary Information

Supplementary Information.
