# Supplementary material for: Chemical hybridization of sulfasalazine and dihydroartemisinin promotes brain tumor cell death
Source: Sci Rep. 2021 Oct 21;11:20766. doi: 10.1038/s41598-021-99960-z (PMC8531376; doi:10.1038/s41598-021-99960-z)
Supplement: Supplementary file 1 — Supplementary Information. [file 41598_2021_99960_MOESM1_ESM.pdf]

Supporting Information

Chemical Hybridization of Sulfasalazine and Dihydroartemisinin Promotes Brain Tumor Cell Death

Annemarie Ackermann[1], Aysun Çapcı[2], Michael Buchfelder[3], Svetlana B. Tsogoeva[2,*] and Nicolai Savaskan[1,3,4,*]

[1]Translational Cell Biology & Neurooncology Lab, Department of Neurosurgery, Universitätsklinikum Medical School Erlangen, Friedrich Alexander University of Erlangen – Nürnberg (FAU), Erlangen, Germany; [2]Organic Chemistry Chair I and Interdisciplinary Center for Molecular Materials (ICMM), Friedrich-Alexander University of Erlangen-Nürnberg, Nikolaus Fiebiger-Straße 10, 91058 Erlangen, Germany; [3]Department of Neurosurgery, Universitätsklinikum Medical School Erlangen, Friedrich Alexander University of Erlangen – Nürnberg (FAU), Erlangen, Germany; [4]BiMECON Ent. Berlin, Germany

Table 1S. Properties of investigated parent compounds and hybrid **AC254**.

Compound	Molecular Formula	MW (g/mol)	Melting Point	Rf[c]	Color	Purity[d]
**Dihydroartemisinin**	$C_{27}H_{29}NO_{11}$	284.35	164-165 °C [a]	0.46	White crystalline powder	+
**AC254**	$C_{33}H_{36}N_4O_9S$	664.73	148-150 °C	0.24	Yellow powder	+
**SAS**	$C_{18}H_{15}N_4O_5S$	398.39	220 °C [b]	-	Yellow powder	+

[a] Ref. 1. [b] Ref. 2 [c] R_f values were determined in the solvent mixture of hexane : ethylacetate (6:4) [d] DHA and SSZ were commercially supplied > 95 grade. Purity of hybrid was confirmed by Elemental Analysis.

1. National Center for Biotechnology Information. PubChem Compound Summary for CID 3000518. https://pubchem.ncbi.nlm.nih.gov/compound/3000518. Accessed Sept. 11, 2020.

2. National Center for Biotechnology Information. PubChem Compound Summary for CID 5339, Sulfasalazine. https://pubchem.ncbi.nlm.nih.gov/compound/Sulfasalazine. Accessed Sept. 11, 2020.
